# Broccoli Improves Lipid Metabolism and Intestinal Flora in Mice with Type 2 Diabetes Induced by HFD and STZ Diet

**DOI:** 10.3390/foods13020273

**Published:** 2024-01-15

**Authors:** Xin Li, Zifan Cai, Feiyu Yang, Yunfan Wang, Xinyi Pang, Jing Sun, Xiangfei Li, Yingjian Lu

**Affiliations:** 1 College of Food Science and Engineering, Nanjing University of Finance and Economics, Collaborative Innovation Center for Modern Grain Circulation and Safety, Nanjing 210023, China; 1120201057@stu.nufe.edu.cn (X.L.); 1120211107@stu.nufe.edu.cn (Z.C.); 1220180745@stu.nufe.edu.cn (Y.W.); pangxinyi@nufe.edu.cn (X.P.); jingsun@nufe.edu.cn (J.S.); yingjianlu@nufe.edu.cn (Y.L.); 2 Priority Academic Program, Development of Jiangsu Higher Education Institutions (PAPD), Nanjing 210023, China; 3 College of Food Science and Technology, Nanjing Agricultural University, Nanjing 210095, China; 2023208017@stu.njau.edu.cn

**Keywords:** type 2 diabetes mellitus, insulin sensitivity, inflammation factors, SCFAs, intestinal flora

## Abstract

Globally, type 2 diabetes (T2DM) is on the rise. Maintaining a healthy diet is crucial for both treating and preventing T2DM.As a common vegetable in daily diet, broccoli has antioxidant, anti-inflammatory and anticarcoma physiological activities. We developed a mouse model of type 2 diabetes and carried out a systematic investigation to clarify the function of broccoli in reducing T2DM symptoms and controlling intestinal flora. The findings demonstrated that broccoli could successfully lower fasting blood glucose (FBG), lessen insulin resistance, regulate lipid metabolism, lower the levels of TC, TG, LDL-C, and MDA, stop the expression of IL-1β and IL-6, and decrease the harm that diabetes causes to the pancreas, liver, fat, and other organs and tissues. Furthermore, broccoli altered the intestinal flora’s makeup in mice with T2DM. At the genus level, the relative abundance of *Allobaculum* decreased, and that of *Odoribacter* and *Oscillospira* increased; At the family level, the relative abundances of Odoribacteraceae, Rikenellaceae and S24-7 decreased, while the relative abundances of Erysipelotrichaceae and Rikenellaceae increased.

## 1. Introduction

Diabetes is a chronic disease, with about 90% of people suffering from type 2 diabetes (T2DM). The disease has a high incidence, a variety of causes, and repeated attacks [1]. The aggravation of T2DM can lead to a series of complications such as oral disease, amputation, chronic kidney disease, retinopathy, cardiovascular disease (stroke [2], acute myocardial infarction [3], heart failur [4] and atherosclerosi [5], etc.), neuropathy and osteoporosis, and even coma or even death in severe cases. T2DM is now a significant worldwide public health issue [6]. Currently, there is no cure for diabetes, and patients can alleviate the disease by taking drugs. However, long-term medication will lead to a series of side effects [7], so adopting a natural, safe, mild and non-toxic diet is of great significance in the early prevention and subsequent adjuvant treatment of diabete [8].

Broccoli is a cruciferous vegetable with a high concentration of glucosinolates (compared to other vegetables) and a glycemic index (GI) of 15. Glucosinolates can be converted into sulforaphane (SFN) and participate in body metabolism [9]. SFN has anti-cancer [10], anti-tumor [11], anti-diabetes [12], anti-osteoporosis [13], anti-oxidation [14] and other physiological activities, SFN can alleviate diseases such as chronic obstructive pulmonary disease [15], asthma [16], autism [17] and diabetes complications [18]. Prior research has demonstrated that broccoli can lessen the heart dysfunction caused by diabetes, relieve myocardial hypertrophy and fibrosis [19], and secondly, broccoli can change oxidative stress and inflammatory response [20]. In addition, broccoli can also regulate lipid metabolism and reduce density lipoprotein concentration [21]. Many in vitro experiments, animal experiments and clinical trials have shown that broccoli metabolites have a potential positive correlation with maintaining health, and long-term consumption of broccoli can lower the risk of developing chronic illnesses such diabetes, obesity, heart disease, and brain disorders [22].

Intestinal flora is the microbial community that is adapted to the anaerobic environment throughout the gastrointestinal system of animals, and the composition of intestinal flora is different in different species. The number of mammalian intestinal flora is about 10^12^, and the identified strains can be divided into 9 phylum according to species (Firmicutes, Bacteroidetes, Proteobacteria, Actinomyces, *Micrococcus*, *Verrucomicrobia*, *Clostridium*, Cyanobacteria, *Spirochaeta* and VadinBE97), more than 50 species of genus (Allobaculum, Odoribacter, Oscillospira, etc.), 800–1000 species [23]. The degree to which the host’s growth rate, material metabolism, and immune system are impacted depends on the variety and richness of its gut flora [24], among which Bacteroides and Firmicutes account for about 90% of the total intestinal flora and are closely related to health [25]. The composition of intestinal flora is not only affected by species, but also closely related to dietary habits and growth environment. Some vegetables (such as broccoli, carrot, celery, pumpkin, etc.), fruits (such as apples, bananas, kiwi, etc.) and beans (lentils, peas, black beans, etc.) in the daily diet are rich in dietary and some polysaccharides that cannot be absorbed and utilized by the host are selectively absorbed and utilized by the intestinal flora, which can sustain the host’s health and encourage the growth of advantageous microorganisms [26].

In this study, we first evaluated at how broccoli affected the body weight, blood glucose level, and organ weight of mice given STZ-induced diabetes. We also evaluated at how broccoli juice affected organ histology, inflammatory factor expression levels, and lipid metabolism. Lastly, we collected samples of mouse feces and used GC-MS and 16S rRNA macro gene sequencing to quantify the changes in intestinal microbial species and metabolites. This allowed us to determine the specific contribution of broccoli to the improvement of diabetes.

## 2. Materials and methods

### 2.1. Animals and Experimental Design

**Figure 1:**
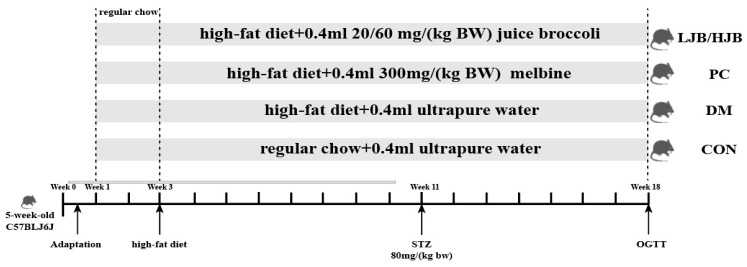
Diet and intragastric samples of mice in different group. CON: Mice fed with normal diet and administered sterile water via gavage.DM: Mice fed with HFD and administered sterile water via gavage. PC: Mice fed with HFD and administered 300 mg (kg BW)^−1^d^−1^ metformin via gavage. LJB: Mice fed with HFD and administered 20 mg (kg BW)^−1^d^−1^ juiced broccoli via gavage. HJB: Mice fed with HFD and administered 60 mg (kg BW)^−1^ d^−1^ juiced broccoli via gavage. The images of H&Estained tissues were viewed under a light microscope at 400× magnification.

Fifty five-week-old male C57BL/6J mice weighed 25 ± 1.8 g. Mice were separated into 5 groups, each consisting of 10 mice, and were acquired from Beijing Weihe Laboratory Animal Technology Co., LTD. (SCXK(JING) 2016-0011, Beijing, China). They were maintained housed in groups of four mice per cage, at temperature (22 °C ± 2 °C) and humidity (55% ± 5%) with 12:12 hlight/dark cycle, and in groups of four mice per cage. Ethical approval for this study obtained from the Animal Ethics Committee of Nanjing Agricultural University, China (SYXK 2017-0007). After 10 days of adaptation, experimental groups and respective treatments are illustrated in Figure 1.

From week 1 to week 3, all mice were fed a normal diet to adapt to the new environment. After week 3, all mice were randomly divided into 5 groups (10 in each group), which were as follows: (1) control (CON) group: standard chow diet, with 0.4 mL ultrapure water intragastric administration; (2) Type 2 diabetes (DM) group: high-fat diet (H10060, HFK bioscience, Beijing, China), with 0.4 mL ultrapure water intragastric administration; (3) positive control (PC) group: high-fat diet, with 0.4 mL 300 mg/(kg BW) melbine intragastric administration; (3) Positive control (PC) group: High-fat diet, with 0.4 mL 300 mg/(kg BW) Melbine intragastric Administration; (4) LJB group: high-fat diet, with 0.4 mL 20 mg/(kg BW) juiced broccoli intragastric administration; (5) HJB group: high-fat diet, with 0.4 mL 60 mg/(kg BW) juiced broccoli intragastric administration. From the first day of week 4 to the end of week 18, mice in CON group were still fed conventional diet, while mice in DM group and PC group were fed high-fat diet (H10060, HFK bioscience, Beijing, China). Mice in LJB group and HJB group were fed high-fat diet and injected 0.4 mL broccoli juice (the LJB group received 20 mg (kg BW)^−1^d^−1^ and the HJB group received 60 mg (kg BW)^−1^d^−1^). Streptozotocin (STZ) (Sigma, St. Louis, MO, USA) was injected into DM group, PC group, LJB group and HJB group starting from day 1 of the 11th week, and all mice were given intraperitoneal injection after fasting for 12 h. Mice in CON group were intrabitoneally injected with 50 mmol L^−1^ citrate buffer (pH 4.5), STZ, dissolved in 50 mmol L^−1^ citrate buffer, was injected intrabitonally into the DM, PC, LJB, and HJB groups’ subjects at a dose of 80 mg (kg BW)^−1^d^−1^. Additionally, STZ injections were maintained until the conclusion of week 18 in order to create a type 2 diabetes model.

### 2.2. Chemicals and Reagen

Broccoli was purchased from a local store in Nanjing city, Jiangsu Province, China, then add ultra-pure water for juiced, the ACCU-CHEK glucometer were obtained from Roche (Mannheim, Germany); Human insulin was obtained from Novo Nordisk (Copenhagen, Denmark); Beijing Solarbio Biological Technology Co., Ltd. provided the metformin, while Nanjing Jiancheng Bioengineering Institute provided the commercial kits (Nanjing, China).

### 2.3. Mice Body Weight and Organ Indexes

All mice were killed after an 18-week experiment, following a 12-h fast. Body weight was measured weekly and before sacrifice. Liver, kidney, spleen, fat and pancreas were collected after sacrifice. The organ index was computed utilizing the subsequent formula:

(1) $$\mathrm{Organ}\;\mathrm{index}\;=\;\frac{\mathrm{organ}\;\mathrm{mass}\;(\mathrm{mg})}{\mathrm{animal}\;\mathrm{body}\;\mathrm{mass}\;(g)}$$

### 2.4. Oral Glucose Tolerance Test (OGTT) and Insulin Tolerance Test (ITT)

At the 18th week of the trial, mice fasted for 12 h were given an oral glucose tolerance test (IGTT) with no restriction of water intake during the fast. Glucose was injected intraperitoneally at a dose of 2 g (kg BW)^−1^. Using the ACCU-CHEK glucose meter, blood glucose was measured at 0 (the instantaneous time before glucose injection), 30, 60, and 120 min.

At week 18 of the study (3 days before the sacrifice), mice that had fasted for 5 h were tested for insulin tolerance (ITT) and drank water freely during the fast. Insulin was injected intraperitoneally at a concentration of (0.75 U/kg BW). The ACCU-CHEK glucose meter was used to measure blood glucose at 0 min (before glucose injection), 30 min, 60 min, 90 min, and 120 min.

### 2.5. Biochemical Measurements

Using an ELISA kit, the fasting insulin (FINS) was determined. The following is the formula for the homeostatic model assessment of insulin resistance (HOMA-IR) and the insulin sensitivity index (ISI):

(2) $$ISI=ln(\frac{1}{FINS}*FBG)$$

(3) $$HOMA-IR=FINS*\frac{FBG}{22.5}$$

Trunk blood were collected from the eye socket vein, and centrifuged (3000× *g*, 10 min) to obtain serum for the biochemical measurements. Serum total cholesterol (TC), triglycerides (TG), low-density lipoprotein cholesterol (LDL-C), high-density lipoprotein cholesterol (HDL-C) were quantified with the com-mercial kit (Nanjing Jiancheng Biology Engineering Institute, Nanjing, China).

As directed by the manufacturer, the ELISA kits were used to quantitatively detect tumor necrosis factor-α (TNF-α), interleukin-1β (IL-1β), interleukin-6 (IL-6) and interleukin-10 (IL-10).

Measurements of oxidative stress Using a commercial kit, the following biochemical parameters were measured: lipopolysaccharide (LPS), glutathione (GSH), glutathione peroxidase (GSH-Px), malondialdehyde (MDA), and superoxide dismutase (SOD). and the level were measured in accordance with their particular protocols.

### 2.6. Histopathological Observation of Mice Epididymal White Adipose, Pancreas, Kidney, Ileumand and Liver

A small portion of the epididymal white adipose, pancreas, kidney, ileumand and liver was excised cut off and fixed perserved in 10% formalin. Shanghai Erwan Biotechnology Co., Ltd. (Shanghai, China) completed out the histopathological work.

### 2.7. Determination Content of Short-Chain Fatty Acids and Intestinal Flora

After the mice were fed for 18 weeks, under aseptic conditions fecal pellets of mice were freshly collected, instantly frozen with dry ice, and then stored at −80 °C. The Beijing Genomics Institute completed tests on the intestinal flora, collected microbiome DNA from 100 mg of fecal samples, and amplified the V3–V4 area of 16S RNA using PCR. Before the analysis, the PCR products underwent processing and purification. A gas chromatograph-mass spectrometer (GC-MS) was used to measure the amount of short-chain fatty acids (SCFAs), which include acetic acid, propionic acid, butyric acid, valeric acid, isovaleric acid, and total short-chain fatty acids.

### 2.8. Statistical Analysis

All experiments were run three times. Every outcome is displayed as the means ± SEM. Software from GraphPad, namely Prism 9.5.1, was used in every instance to handle and analyze statistical data (GraphPad Software). To determine whether the difference between the five groups had statistical significance, a one-way analysis of variance (ANOVA) was used. *p* values < 0.05 was deemed statistically significant.

## 3. Results

### 3.1. Impact of Different Broccoli Juice Dosages on Body Weight, Fasting Blood Glucose (FBG), and Organ Indices

**Figure 2:**
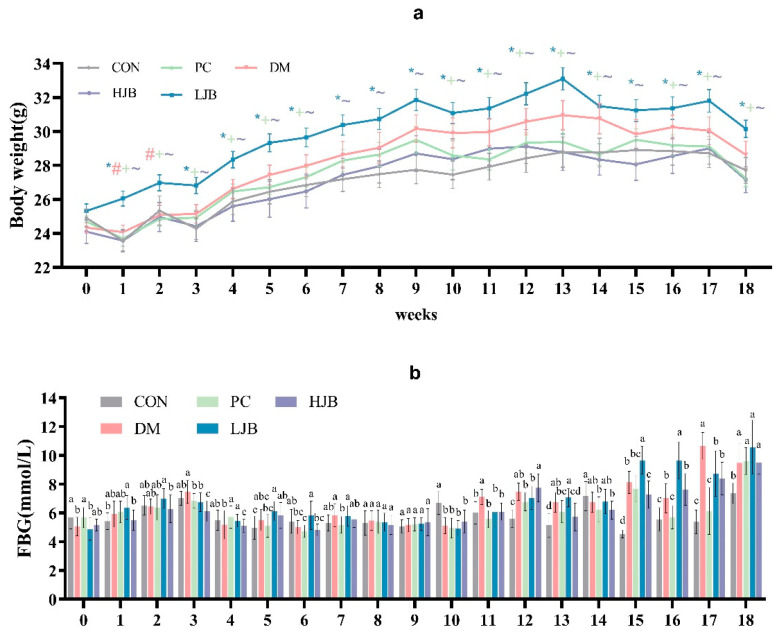
Impact of varying broccoli juice dosages on (**a**) body weight and (**b**) fasting blood glucose (FBG) levels. For ten mice in each group, the values are mean ± SEM. CON: Mice fed with standard chow diet and administered sterile water via gavage.DM: Mice fed with HFD and administered sterile water via gavage. PC: Mice fed with HFD and administered 300 mg (kg BW)^−1^d^−1^ metformin via gavage. LJB: Mice fed with HFD and administered 20 mg (kg BW)^−1^d^−1^ juiced broccoli via gavage. HJB: Mice fed with HFD and administered 60 mg (kg BW)^−1^d^−1^ juiced broccoli via gavage. * *p* < 0.05 LJB group vs. CON group, # *p* < 0.05 LJB group vs. MD group, +*p* < 0.05 LJB group vs. PC group, ~*p* < 0.05 LJB group vs. HJB group.

We examined how the body weight of T2DM mice was affected by HFD and various dosages of juiced broccoli. (Figure 2a, *p* < 0.05). As expected, HFD significantly promoted weight gain in mice before STZ induction, the body weight was significantly higher in the DM and LJB groups than in the CON/PC and HJB groups after 8 weeks of dietary intervention. Furthermore, there were no discernible variations in body weight between the CON/PC and HJB groups; similar results were noted when contrasting the DM and LJB groups (Figure 2a, *p* < 0.05). Throughout the experiment, we observed that the body weights of CON/PC and HJB weren’t significantly different. These results demonstrated high doses of juiced broccoli can reduce weight of the T2DM mice.

During the experiment, we measured and recorded the fasting blood glucose (FBG) of the mice every week. From the results, we can know that there was a significant difference in FBG in the mice. In the third week of the experiment, the FBG of DM group was significantly increased (Figure 2b, *p* < 0.05), however, the FBG of the mice decreased significantly at the fourth week. In addition, FBG was relatively stable in mice from week 4 to week 10 (Figure 2b, *p* < 0.05). At week 11 and 12, FBG in DM group was significantly higher than that in CON group by 18.7% and 33.2%. At week 13, FBG of DM/PC and LJB mice increased significantly compared with CON group by 30.7%, 18.7% and 37.3%, respectively (Figure 2b, *p* < 0.05), there was no significant difference between HJB group and DM group. At week 14, FBG was significantly lower in the PC and LJB groups than in the other groups. At week 15–17, FBG was increased in all four groups compared with the CON group, but the increase of FBG in PC and LJB groups was smaller than that in CON group. At week 18, mice in the CON group had less FBG, compared with CON group, DM, PC, LJB and HJB groups increased by 28.8%, 30.5%, 43.7% and 29.0%, respectively, and there was no significant difference in FBG between the other four groups. These results suggest that HFD and T2DM can cause glucose intolerance, while high-dose juiced broccoli can improve glucose metabolism and lower blood sugar in mice.

**Figure 3:**
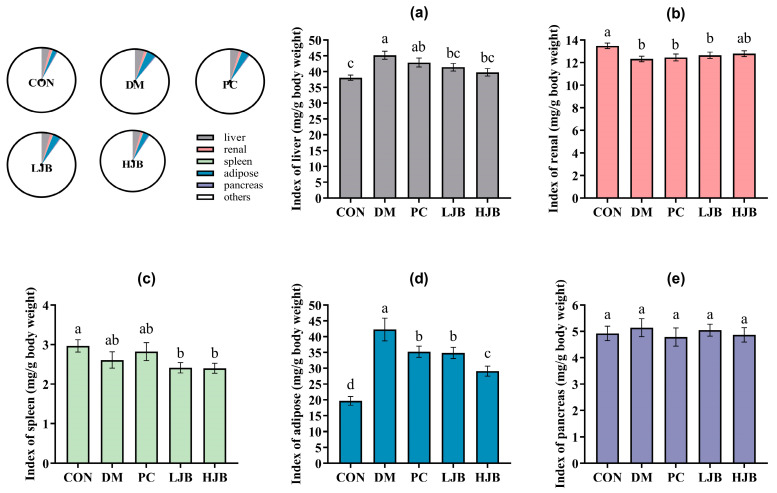
Organ indexes determined for various mice groups. (**a**) The liver index. (**b**) The renal index. The spleen index (**c**). (**d**) The Adipose Index. (**e**) The pancreatic index. CON: Mice fed with standard chow diet and administered sterile water via gavage.DM: Mice fed with HFD and administered sterile water via gavage. PC: Mice fed with HFD and administered 300 mg (kg BW)^−1^d^−1^ metformin via gavage. LJB: Mice fed with HFD and administered 20 mg (kg BW)^−1^d^−1^ juiced broccoli via gavage. HJB: Mice fed with HFD and administered 60 mg (kg BW)^−1^d^−1^ juiced broccoli via gavage.

Next, to check the effect of juice broccoli on organ and tissues we also measured the weight of liver/renal/spleen/adipose and pancrease tissues of T2DM mice. We found that the DM and PC groups had higher index of liver than the CON group, DM increased by 18.7% and PC increased by 12.6%. The liver index was still considerably lower in the HJB and LJB groups compared to the DM group by 11.9% and 8.4%, respectively. The organ weights of the PC group and DM group did not differ significantly (Figure 3a, *p* < 0.05). Additionally, the renal index was 6.2%, 7.6%, and 8.6% lower in the DM/PC and LJB groups than in the CON group (Figure 3b, *p* < 0.05).

The results of masurement of spleen show that compared with CON group, index of LJB and HJB Groups had significantly decreased by 18.7% and 19.0% (Figure 3c, *p* < 0.05). Furthermore, the CON group had a substantially higher level of adipose than the other four groups: the DM group had an increase of 114.6%, the PC group had a 79% rise, the LJB group had a 76.9% increase, and the HJB group had a 47.9% increase. The adipose indexes of the PC, LJB, and HJB groups fell by 16.7%, 17.8%, and 31.1%, respectively, in comparison to the DM group (Figure 3d, *p* < 0.05). The results from the weight of pancrease demonstrate that the various mice groups did not differ significantly from one another (Figure 3e, *p* > 0.05). When combined, these findings imply that T2DM mice administered DMBG or broccoli juice can decrease their adipose tissue weight.

### 3.2. Impact of Different Juice Broccoli Dosages on OGTT and ITT

**Figure 4:**
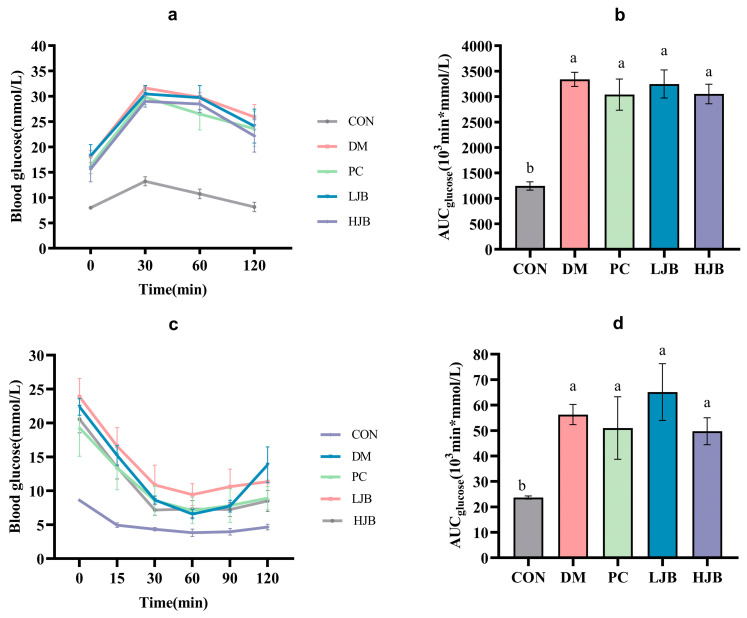
Impact of different juice broccoli dosages on the following tests: (**a**) insulin tolerance test (ITT) results; (**b**) OGTT AUC; (**c**) oral glucose tolerance test (OGTT) results; and (**d**) ITT AUC. CON: Mice fed with standard chow diet and administered sterile water via gavage.DM: Mice fed with HFD and administered sterile water via gavage. PC: Mice fed with HFD and administered 300 mg (kg BW)^−1^d^−1^ metformin via gavage. LJB: Mice fed with HFD and administered 20 mg (kg BW)^−1^d^−1^ juiced broccoli via gavage. HJB: Mice fed with HFD and administered 60 mg (kg BW)^−1^d^−1^ juiced broccoli via gavage.

Figure 4a,b shows the blood glucose curves for each group following the oral glucose tolerance test (OGTT) and AUC. Compared to the CON group, the blood glucose and AUC were considerably higher in the DM, PC, LJB, and HJB groups. The AUC for the groups DM, PC, LJB, and HJB was found to be 168.4%, 144.3%, 161.1%, and 145.3% greater than that of the CON group, in that order. Following the 30-min glucose treatment (2 g/kg BW), the mice’s blood sugar levels reached their peak in all groups; compared to the CON group, the blood glucose levels of the DM, PC, LJB, and HJB groups increased significantly (Figure 4a, *p* < 0.05). At the end of OGTT (120 min), there was no significant difference between CON group and 0 min, but the blood glucose level in DM group, PC group, LJB group and HJB group was 43.2%, 44.3%, 32.2% and 41.8% higher than that at 0 min, respectively.

The blood glucose curves of each group following of the insulin tolerance test (ITT) and AUC are shown in Figure 4c,d. During the whole experiment. The mice in the DM group, PC group, LJB group, and HJB group had considerably greater blood glucose levels (Figure 4c, *p* < 0.05) and an AUC (Figure 4d, *p* < 0.05) than the mice in the CON group. The AUC of DM group, PC group, LJB group and HJB group was 137.6%, 115.2%, 174.9% and 109.9% higher than that of CON group, respectively. After inadministration of insulin (0.75 U /kg BW), the blood glucose level of HJB group was reduced to the minimum value after 30 min, and that of the other four groups was reduced to the minimum value after 60 min. The minimum blood glucose level of the other four groups was 71.7%, 85.9%, 147.1% and 87.4% higher than that of the CON group, respectively. At the end of ITT (120 min), in every group, the mice’s blood glucose level was considerably lower than it was at 0 min.

### 3.3. Impact of Different Broccoli Juice Doses on Insulin Sensitivity

**Figure 5:**
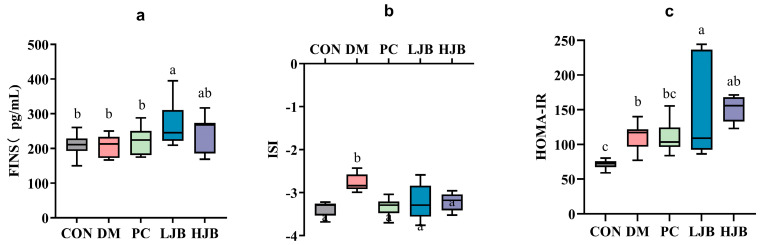
Impact of different juice broccoli doses on (**a**) the fasting insulin (FINS), (**b**) the insulin sensitivity index (ISI), (**c**) the homeostatic model assessment of insulin resistance (HOMA-IR). CON: Mice fed with standard chow diet and administered sterile water via gavage.DM: Mice fed with HFD and administered sterile water via gavage. PC: Mice fed with HFD and administered 300 mg (kg BW)^−1^d^−1^ metformin via gavage. LJB: Mice fed with HFD and administered 20 mg (kg BW)^−1^d^−1^ juiced broccoli via gavage. HJB: Mice fed with HFD and administered 60 mg (kg BW)^−1^d^−1^ juiced broccoli via gavage.

Before the trial’s conclusion, we examined the effects of T2DM on the FINS, ISI, and HOMA-IR of mice following 6 weeks of dietary and STZ intervention in order to throw light on the effects of various dosages of juice broccoli on insulin sensitivity. As shown in Figure 5a, although there were no appreciable variations in the FINS between the CON/DM/PC and HJB groups, the FINS was much greater in the LJB group. At the ISI, DM group’s ISI level increased by 19.3%, which is significant from CON group’s level. ISI (Figure 5b, *p* < 0.05) in PC, LJB and HJB groups were lower and had no significant difference from those in CON group. Compared with DM group, ISI in PC, LJB and HJB groups were decreased by 10.4%, 9.8% and 9.2%, respectively (Figure 5b, *p* < 0.05). During the experiment, the insulin resistance was assessed by HOMA-IR (Figure 5c, *p* < 0.05). The results showed that the LJB, DM, and HJB groups had higher insulin resistance than the CON group.

### 3.4. Impact of Different Broccoli Juice Dosages on Serum Lipid, Inflammation Factors and Oxidative Stress

**Figure 6:**
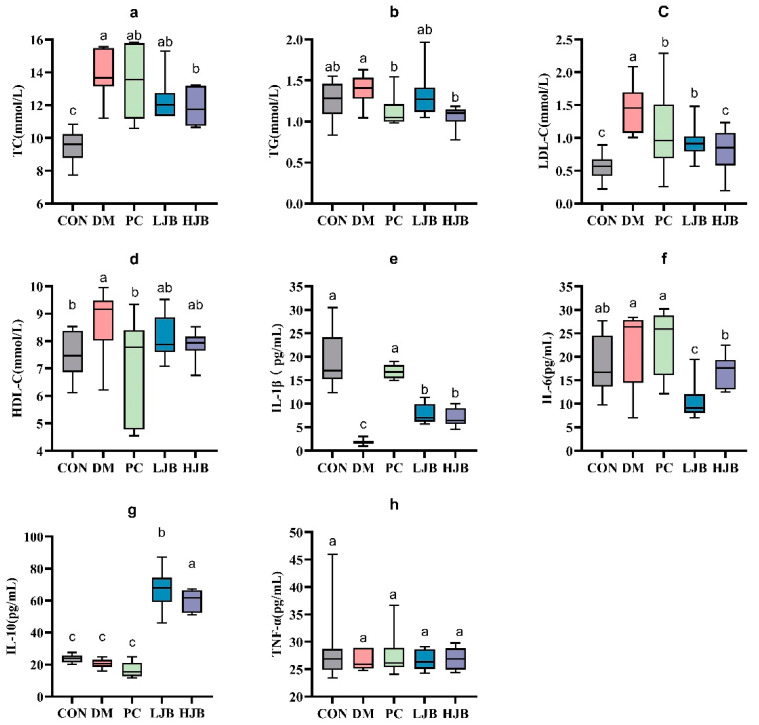
Impact of different juice broccoli dosages on serum lipid concentrations. (**a**) total cholesterol (TC), (**b**) triglycerides (TG), (**c**) low-density lipoprotein cholesterol (LDL-C), (**d**) high-density lipoprotein cholesterol (HDL-C), (**e**) IL-1β, (**f**) IL-6, (**g**) IL-10 and (**h**) TNF-α. CON: Mice fed with standard chow diet and administered sterile water via gavage.DM: Mice fed with HFD and administered sterile water via gavage. PC: Mice fed with HFD and administered 300 mg (kg BW)^−1^d^−1^ metformin via gavage. LJB: Mice fed with HFD and administered 20 mg (kg BW)^−1^d^−1^ juiced broccoli via gavage. HJB: Mice fed with HFD and administered 60 mg (kg BW)^−1^d^−1^ juiced broccoli via gavage.

We examined the levels of TC, TG, LDL-C, and HDL-C in order to clarify the effects of different doses of juiced broccoli on lipid metabolism in diabetic mice. As shown in Figure 6. We can see from the test data diabetic significantly promoted TC gain in mice, DM, PC, LJB and HJB groups were 44.1%, 38.6%, 30.5% and 25.1% higher than CON group, respectively. Compared with DM group, PC group, LJB group and HJB group were reduced by 12.5%, 30.8% and 43.1%, respectively. Juiced broccoli was shown to be an effective way to lower the cholesterol content in diabetic mice (TC in the HJB group was 17.8% lower than in the LJB group), and the high dose (60 mg/kg BW) had a greater effect. We found that the TG levels in the PC group and HJB group were considerably lower—by 19.5% and 23.6%, respectively—than in the DM group (Figure 6b, *p* < 0.05).

Furthermore, determination of LDL-C indicated that compared with CON group, LDL-C content in DM group, PC group and LJB group was significantly increased, by 158.6%, 91.7%, 66.8% and 43.9%, respectively (Figure 6c, *p* < 0.05). Compared with DM group, LDL-C content in the other four groups was significantly decreased, which was 61.3%, 25.9%, 35.5% and 44.3% in CON group, PC group, LJB group and HJB group, respectively, There was no difference in the LDL-C content between the PC group and the LJB group, nor between the HJB group and the CON group. Next, HDL-C data analysis results suggested that compared with CON group, HDL-C content in DM group was significantly increased by 14.4%. Compared with DM group, HDL-C content in PC group was significantly reduced by 18.4% (Figure 6d, *p* < 0.05). These results demonstrated that providing diabetic mice juiced broccoli can significantly lower their blood levels of TC, TG, and LDL-C., and a high dose (60 mg/kg BW) has excellent outcomes.

It is crucial to find out the impact of juiced broccoli on the production of levels of inflammatory markers in addition to its effect on lipid metabolism in diabetic mice. As illustrated in Figure 6e, the IL-1β levels in the four other groups—DM, PC, LJB, and HJB—were significantly lower than those of the CON group, at 90.2%, 11.1%, 58.4%, and 62.0%, respectively.

IL-6 analysis showed that compared with CON group, IL-6 level in LJB group was significantly decreased by 42.9%. Compared with DM group, IL-6 levels in LJB group and HJB group were significantly reduced by 42.9% and 22.5%, respectively. (Figure 6f, *p <* 0.05) Compared with PC group, IL-6 levels in LJB group and HJB group were significantly decreased by 54.5% and 27.4%, respectively. Compared with LJB group, IL-6 level in HJB group was significantly increased by 59.5%. We also measured IL-10 levels in addition to IL-1 β and IL-6, compared with CON group, IL-10 levels in LJB group and HJB group were significantly increased by 182.65% and 152.6%, respectively (Figure 6g, *p* < 0.05). Compared with LJB group, IL-10 level in HJB group was significantly decreased by 10.6%. Overall, our results suggest that juiced broccoli can increase IL-1β level and decrease IL-6 level in diabetic mice.

**Figure 7:**
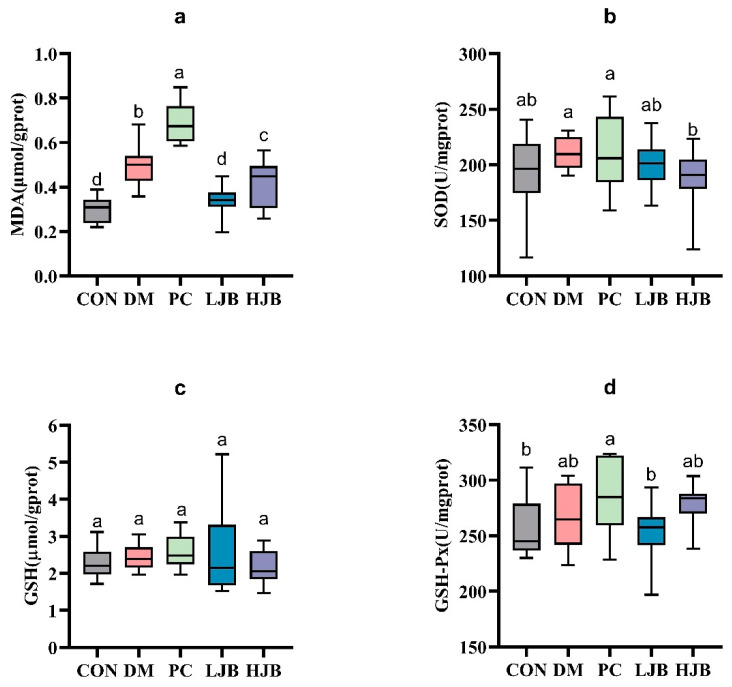
Impact of varying juice broccoli doses on indicators of oxidative stress. (**a**) malonaldehyde (MDA), (**b**) superoxide dismutase (SOD), (**c**) glutathione (GSH) and (**d**) glutathione peroxidase (GSH-Px). CON: Mice fed with standard chow diet and administered sterile water via gavage.DM: Mice fed with HFD and administered sterile water via gavage. PC: Mice fed with HFD and administered 300 mg (kg BW)^−1^d^−1^ metformin via gavage. LJB: Mice fed with HFD and administered 20 mg (kg BW)^−1^d^−1^ juiced broccoli via gavage. HJB: Mice fed with HFD and administered 60 mg (kg BW)^−1^d^−1^ juiced broccoli via gavage.

The levels of MDA, SOD, GSH, and GSH-Px in the liver tissue of diabetic mice were measured in order to evaluate the impact of juiced broccoli on the antioxidant capacity of diabetic mice cells (Figure 7). In comparison to the CON group, we noticed the MDA levels in the DM, PC, and HJB groups had dramatically increased by 64.8%, 128.4%, and 38.4%, respectively. (Figure 7a, *p* < 0.05). The MDA level in the PC group was much higher by 38.6% when compared to the DM group, whereas the MDA level in the LJB and HJB groups was much lower by 31.9% and 16.0%, respectively. (Figure 7a, *p* < 0.05). Furthermore, distinctions in GSH-Px have been found in five mouse groups (Figure 7d, *p* < 0.05), compared with PC group, the concentration of GSH-Px in CON group and LJB group was decreased by 9.7% and 11.1%. These findings suggest that Juiced broccoli can significantly reduce MDA levels in diabetic mice, high doses of juiced broccoli can increase GSH-Px concentration in diabetic mice, and DMBG can significantly increase MDA levels in diabetic mice.

### 3.5. Histopathological Analyses of Epididymal White Adipose, Pancreas, Kidney, Ileum and Liver

**Figure 8:**
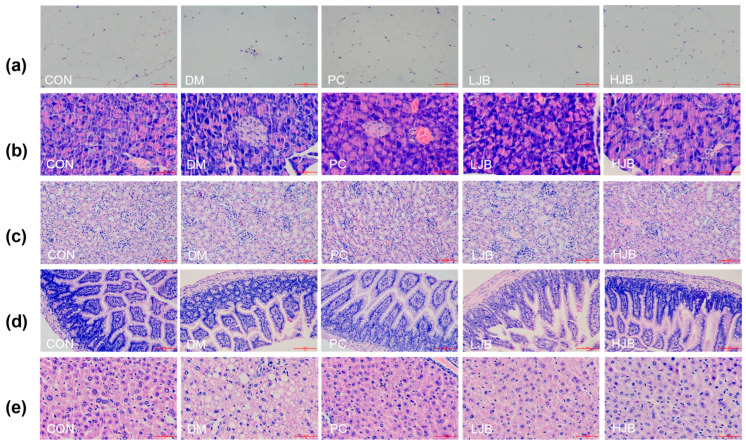
Impact on organ histopathology at different dosages of juice broccoli. (**a**) adipose tissue, (**b**) pancreas tissue, (**c**) kidney tissue, (**d**) ileum tissue, (**e**) liver tissue. CON: Mice fed with standard chow diet and administered sterile water via gavage.DM: Mice fed with HFD and administered sterile water via gavage. PC: Mice fed with HFD and administered 300 mg (kg BW)^−1^d^−1^ metformin via gavage. LJB: Mice fed with HFD and administered 20 mg (kg BW)^−1^d^−1^ juiced broccoli via gavage. HJB: Mice fed with HFD and administered 60 mg (kg BW)^−1^d^−1^ juiced broccoli via gavage. The images of H&E-stained tissues were viewed under a light microscope at 400× magnification. Scale bars are 50 μm.

Hematoxylin and eosin were applied to stain the white adipose tissue of the epididymis, pancreas, kidney, ileum, and liver of five groups of mice in order to explore the effects of broccoli juice on the organ histopathology of T2DM mice (Figure 8).

According to the observation results of adipose tissue sections (Figure 8a), adipose tissue in CON group was uniform in size, neatly arranged and with clear edges. Compared with CON group, part of adipose tissue in DM group and LJB group was significantly enlarged and more disorderly in arrangement. Although the adipose tissue size of PC group did not increase significantly, there was a certain degree of damage at the edge and the shape was not full. There was no significant increase in adipose tissue size in HJB group, and the margin was obvious. Therefore, the effect of high-dose juiced-broccoli (60 mg/kg BW) was better than that of low-dose broccoli (20 mg/kg BW) in alleviating fat cell growth.

As can be seen from the observation of pancreatic tissue sections (Figure 8b), pancreatic cells in CON group were full, evenly distributed and with clear edges. The DM group’s cell count was significantly fewer than that of the CON group, and its margins were unclear as well as to having become atrophic. The number of cells in PC group decreased to some extent, and some cells atrophied. The decrease of pancreatic cell number and cell atrophy in both LJB group and HJB group were alleviated to some extent, and the cell distribution in HJB group was more uniform and the cell morphology was more full.

From the observation results of kidney, ileum and liver tissue sections (Figure 8c–e), we can see that the CON group tissues are closely arranged, and the fat particles in the tissues are small. Compared with CON group, DM group and LJB group had more fat particles, and the order of tissue arrangement was destroyed. The fat particles in PC group and HJB group were reduced to a certain extent, and the order of tissue arrangement was restored to a certain extent.

Altogether, these observations provide further support for juiced broccoli can alleviate and repair the enlargement of fat cells and the damage of pancreas, liver, kidney and ileum tissue to a certain extent, and the effect of high-dose juiced broccoli (60 mg/kg BW) on alleviating the enlargement of fat cells and repairing tissue damage is better than that of low-dose broccoli (20 mg/kg BW).

### 3.6. Impact of Different Juice Broccoli Doses on SCFAs

**Figure 9:**
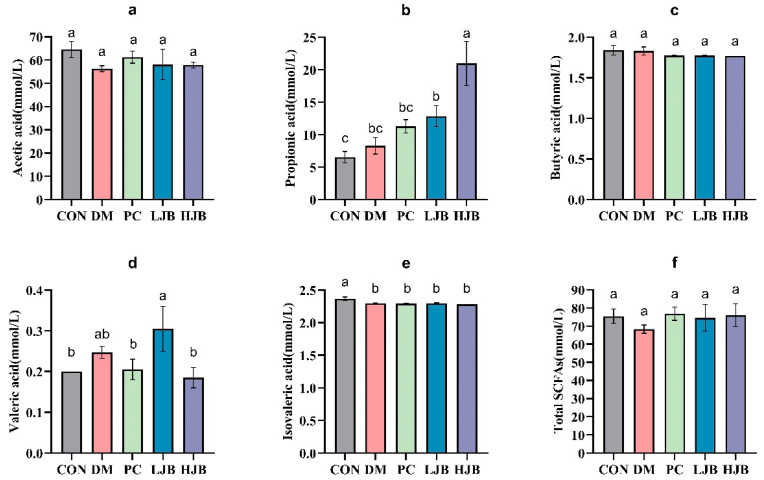
Impact of different juice broccoli doses on SCFAs. (**a**) Acetic acid (**b**) Propionic acid (**c**) Butyric acid (**d**) Valeric acid (**e**) Isovaleric acid (**f**) Total short-chain fatty acids (SCFAs). CON: Mice fed with standard chow diet and administered sterile water via gavage.DM: Mice fed with HFD and administered sterile water via gavage. PC: Mice fed with HFD and administered 300 mg (kg BW)^−1^d^−1^ metformin via gavage. LJB: Mice fed with HFD and administered 20 mg (kg BW)^−1^d^−1^ juiced broccoli via gavage. HJB: Mice fed with HFD and administered 60 mg (kg BW)^−1^d^−1^ juiced broccoli via gavage.

To clarify the effects of juice broccoli on short-chain fatty acid (SCFAs), following an 18-week dietary intervention we used a gas chromatography–mass spectrometer (GC–MS) to determine the amount of SCFAs that inhabit the intestinal tract of diabetic mice. The levels of acetic acid, butyric acid, and total short-chain fatty acids in the intestines of the five groups did not differ significantly, as demonstrated by Figure 9a,c,f.

Compared with CON group, DM group and PC group, the propionic acid concentration in LJB group and HJB group was significantly increased by 96.8% and 221.6%, respectively (Figure 9b, *p* < 0.05). Compared with DM group, LJB group and HJB group were significantly increased by 55.5% and 153.9%, respectively (Figure 9b, *p* < 0.05). Compared with CON group, the concentration of valerate in HJB group was significantly decreased by 5% (Figure 9d, *p* < 0.05). Compared with CON group, the isovaleric acid concentration in DM group, PC group, LJB group and HJB group was significantly decreased by 2.9%, 3.4%, 2.8% and 3.7%, respectively (Figure 9e, *p* < 0.05). In summary, juiced broccoli can increase the concentrations of propionic acid and valeric acid in the intestine, and its regulatory effect is better than DMBG.

### 3.7. Impact of Different Broccoli Juice Doses on Intestinal Flora

**Figure 10:**
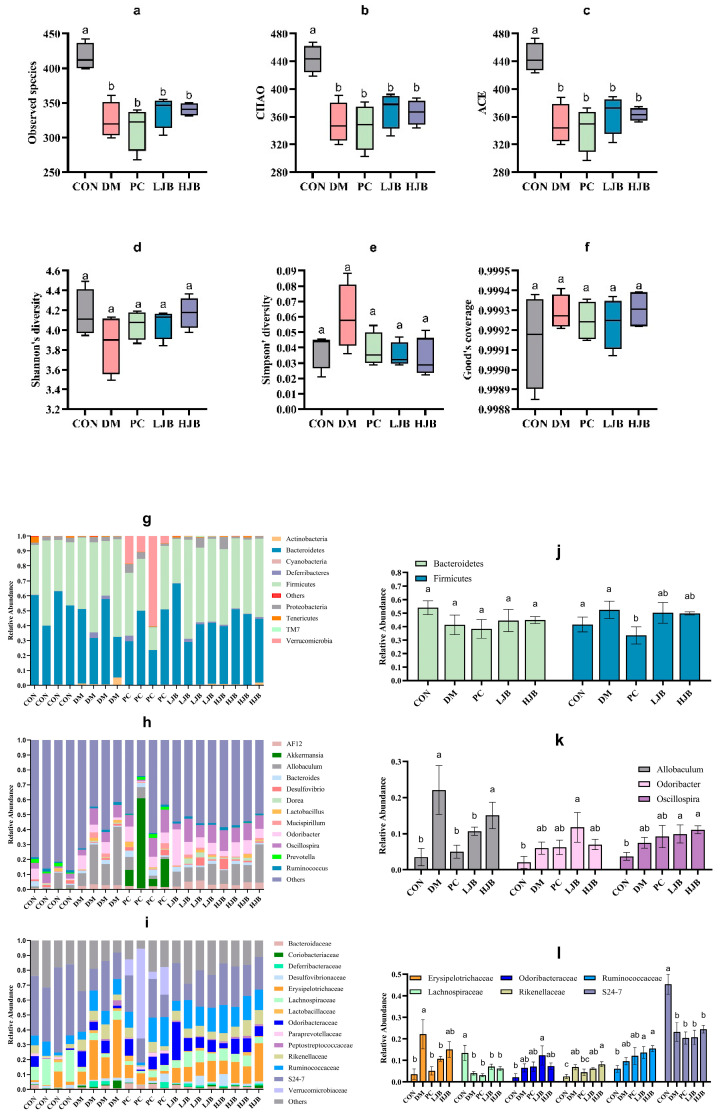
Impact of different broccoli juice doses on the intestinal flora of type 2 diabetic mice. (**a**) Observed species, (**b**) chao index, (**c**) ace index, (**d**) shannon diversity, (**e**) simpson’s diversity, (**f**) good coverage, (**g**) The composition and relative abundance of the gut microbiota at the phylum level, (**h**) The composition and relative abundance of the gut microbiota at the genus level, (**i**) The composition and relative abundance of the gut microbiota at the family level, (**j**) Relative abundance of Firmicutes and Bacteroidetes at the phylum level, (**k**) Relative abundance of *Allobaculum*, *Odoribacter* and *Oscillospira* at the genus level and (**l**) Relative abundance of Erysipelotrichaceae, Lachnospiraceae, Odoribacteraceae, Rikenellaceae, Ruminococcaceae, S24-7 at the family level.

T2DM can lead to imbalance of intestinal ecological environment, especially the low diversity and stability of intestinal flora, to investigate the effects of different doses of juiced broccoli on intestinal flora diversity, we evaluated the α diversity. Observed species, chao, ace, shannon’s diversity, simpson’s diversity and good coverage are shown in Figure 10. Compared with CON group, observed species in DM group, PC group, LJB group and HJB group decreased by 22.0%, 24.7%, 18.9% and 18.2%, respectively (Figure 10a, *p* < 0.05). chao decreased by 20.9%, 22.2%, 16.5% and 17.4% respectively (Figure 10b, *p* < 0.05), ace decreased by 21.5%, 23.2%, 18.2% and 18.4% respectively (Figure 10c, *p* < 0.05). There was no significant difference in shannon’s diversity, simpson’s diversity and good coverage of the five groups of experimental animals. These results indicated that T2DM significantly reduced the abundance of microorganisms in the gut of mice. Adding broccoli to the diet can restore gut flora abundance to some extent.

The taxonomic analysis revealed that juiced broccoli had certain effects on intestinal flora of T2DM mice at the level of phylum (Figure 10g), genus (Figure 10h) and family (Figure 10i). On the phylum levels (Figure 10g). Mice’s gut flora was dominated by Firmicutes and Bacteroides. The relative abundance of firmicutes in the PC group dropped dramatically by 29.3% and 36%, respectively, as compared to the CON group and the DM group. In the intestinal tract of the mice in the CON group, there was a minor amount of Cyanobacteria, but in the intestinal tract of the diabetic mice, it was completely absent. It’s interesting to observe that the relative abundance of Verrucomicrobia in the mice’s guts increased significantly in the DMBG group compared to the CON group, reaching 318 times higher.

On the genus level (Figure 10h), the genera with the dominant relative abundance were *Allobaculum*, *Odoribacter* and *Oscillospira*. In comparison to the CON group, the DM group showed a significantly higher relative abundance of *Allobaculum* (7 times that of the CON group). Additionally, the LJB group demonstrated a significantly higher relative abundance of *Odoribacter* and *Oscillospira* (5.6 and 2.6 times, respectively, of that in the CON group), and the HJB group demonstrated a significantly higher relative abundance of *Allobaculum* and *Oscillospira* (4.3 and 3 times, respectively, of that. Relative *Allobaculum* abundances in the PC and LJB groups were significantly lower (77.3% and 51.7%, respectively) than in the DM group. When the *Allobaculum* relative abundances of the PC and LJB groups were compared to the HJB group, they were significantly higher (3 and 1.4 times less, respectively).

On the family level (Figure 10i), Erysipelotrichaceae, Lachnospiraceae, Odoribacteraceae, Rikenellaceae, Ruminococcaceae and S24-7 were predominant in relative abundance. When comparing the DM group to the CON group, there was a significant increase in the relative abundance of Erysipelotrichaceae and Rikenellaceae (6.2 and 2.7 times, respectively), while there was a significant decrease in the relative fraction of Lachnospiraceae and S24-7 (70% and 48.7%, respectively). The relative abundances of Lachnospiraceae and S24-7 in the PC group were significantly lower than those of the CON group, declining by 76.9% and 55%, respectively. When comparing the LJB group to the CON group, there was a substantial rise in the relative abundance of Ruminococcaceae and Rikenellaceae (2.4 and 2.3 times, respectively), but there was a significant drop in the relative abundance of Lachnospiraceae and S24-7 (47.6% and 54.3%, respectively). The relative abundances of Ruminococcaceae and Rikenellaceae in the HJB group were significantly higher than in the CON group (3.2 and 2.5 times, respectively). Erysipelotrichaceae relative abundances in the PC and LJB groups were much lower (76.9% and 51.7%, respectively) than in the DM group. In comparison to the PC group, the HJB group exhibited a notable increase in the relative abundance of Rikenellaceae, which was 1.8 times higher.

## 4. Discussion

In this study, the effects of different doses of juiced broccoli on T2DM mice were studied from the aspects of lowering blood glucose, lowering blood lipids, reducing inflammation and anti-oxidation, improving organ tissue damage and regulating intestinal flora composition and metabolism. The results showed that broccoli could not only effectively improve the disorder of glucose and lipid metabolism in diabetic mice, but also regulate intestinal flora.

In this study, type 2 diabetic mice fed with a HJB for 18 weeks following a free feeding strategy appeared to show significantly lower increases in FBG to the CON group. This observation is similar to a recent study demonstrating that treated rat liver cancer cells with the main active ingredient (SFN) in broccoli, which showed a significant and dose-dependent decrease in glucose levels in the cells [19]. Juiced broccoli is a natural and side-effect free food that plays an important role in lowering FBG. Juiced broccoli can treat T2DM by improving insulin secretion and insulin resistance in mice, FINS and ISI in mice treated with juiced broccoli are significantly reduced. Previously, Other researchers found that SFN reduced fasting blood sugar in rats by 7.5%, but significantly increased insulin sensitivity and glucose tolerance, however, the subsequent trials conducted by this team in T2DM patients found that SFN had no significant effect on HOMA-IR and ISI [27]. Bahaddoran et al. found that broccoli sprouts can significantly reduce the serum insulin concentration and HOMA-IR of patients [28]. According to the research of Axelsson et al., SFN, the active ingredient of broccoli, can reduce the production of glucose by down-regulating the key gluconogenic enzyme and increasing insulin sensitivity through NRF2, thus reducing blood sugar [27] Cho et al. found that broccoli extract can reduce the expression of glycated protein, this is related to the effect of broccoli on lowering blood sugar [29]. Many studies have shown that the potential role of broccoli in reducing insulin resistance is related to SFN. Studies have shown that SFN can activate AKT, the intermediate of insulin signal transduction, but islet secretion is also affected by many other pathways [30], and its specific mechanism needs further study.

Type 2 diabetes occurrence involves changes in many metabolism related to serum lipid, inflammation factors and oxidative stress. In this study, we found that the juiced broccoli intervention can improve the lipid metabolism of type 2 diabetic mice, especially in the HJB group. High-dose juiced broccoli can significantly reduce the contents of serum TC, TG and LDL-C. The previous mechanism investigation revealed that the increase of TC, TG and LDL-C is closely related to the risk of cardiovascular disease (CVD) leading to death in T2DM patients [31], juiced broccoli may have a positive effect on T2DM mice by regulating lipid metabolism. The research results of Laura et al. proved that broccoli can reduce the cholesterol content in hamsters, and the metabolism of cholesterol and lipids may be different depending on the sex of the animals. Paul et al. found that broccoli extract can not only up-regulate oxidase coding base, but also down-regulate the coding base of proteins related to fatty acid synthesis and transportation, and the occurrence of lipid oxidation can reduce the weight of fat in the liver [32]. The specific mechanism of broccoli’s influence on lipid metabolism still needs to be further studied.

In this study, we also found that juiced broccoli reduces levels of cellular inflammatory factors (IL-1β and IL-6). Chronic inflammation is a common feature of T2DM patients, and the increased concentration of cellular inflammatory factors may lead to cardiovascular diseases and metabolic syndrome [33]. IL-1β can activate pro-inflammatory mediators and infiltrate macrophages, cause insulin resistance and oxidative stress, and lead to β cell dysfunction and impaired insulin secretion [34]. Increased levels of IL-6 lead to decreased expression of genes involved in adiponectin production, adiponectin is a adipose-specific plasma protein, it can enhance insulin sensitivity [35], anti-inflammatory [36], and increase the concentration of HDL-C [33]. Juicer Broccoli reduces the levels of IL-1β and IL-6 in cells, which helps to alleviate the decrease of insulin secretion and insulin resistance caused by T2DM, improve the disorder of glucose metabolism, reduce inflammatory response and cardiovascular disease risk [37]. We determined four oxidative stress-related substances (MDA, SOD, GSH and GSH-PX) in mice, and found that juiced broccoli can significantly reduce MDA levels in T2DM mice. MDA is one of the markers of lipid peroxidation [38], becoming the main cause of diabete-related complications [39]. In agreement with our result, Bahadoran et al. found that broccoli bud powder significantly reduced serum MDA content by 9% in T2DM patients [40].

Adipose tissue can produce adipokines and exosomes, which are involved in the regulation of many important physiological functions [41]. The increase of adipose tissue in DM mice affects systemic metabolic homeostasis and leads to systemic insulin resistance [42] and inflammation [43]. According to the results of adipose tissue section and adipose index test, high-dose juiced broccoli can significantly improve the growth of adipocytes and reduce body adipose index. Juiced broccoli can also relieve islet cell damage caused by T2DM [44]. Kidney is an important organ for metabolite production, endocrine regulation and reabsorption in the body. Glucose metabolism disorder and inflammatory response caused by diabetes will cause damage to the kidney [44]. Liver is an important organ that regulates carbohydrate and lipid metabolism [45]. The ileum is an important place of absorption and secretion in the body. Combined with the organ index and histological observation results, it can be seen that juiced broccoli can significantly reduce the liver index of T2DM mice, reduce the fat content in kidney, liver and ileum, and alleviate insulin resistance. Sun et al. [46] also found that high fat significantly increased liver index in mice.

Short-chain fatty acids (SCFAs) are the main metabolites of carbohydrate after fermentation by intestinal flora. The production of SCFAs can inhibit the growth of pathogenic microorganisms, improve intestinal microenvironment [47], regulate host blood sugar and lipid metabolism, promote nutrients, improve insulin sensitivity, inhibit inflammation and tumor cell invasion and metastasis. It is closely related to health [48]. From the results, we know that juiced broccoli can significantly increase the concentrations of propionic acid and valeric acid in T2DM mice. Propionic acid can play a role in regulating the metabolism of carbohydrate and lipid substances, anti-inflammation and reducing endotoxin [49]. Valeric acid can lower arterial blood pressure and prevent cardiovascular and cerebrovascular diseases [47]. In addition, previous studies have shown that dietary fiber can increase the content of butyric acid in mice [50], and hawthorn polysaccharide can increase the content of acetic acid and propionic acid in the intestines of mice [51]. The combination of multiple diets will be more beneficial to diabetic patients.

Intestinal flora affects a variety of physiological and biochemical processes such as micronutrient synthesis, pathogen defense, carbohydrate and lipid metabolism and immunity in the host body [52]. The composition of intestinal flora is closely related to obesity, insulin resistance and T2DM development [53]. Our study results showed that there were significant differences in the abundance and diversity of intestinal flora between T2DM mice and normal mice, and Observed species, chao and ace of DM group, PC group, LJB group and HJB group were significantly reduced, indicating that diabetes significantly decreased the abundance of intestinal flora and affected the homeostasis of intestinal environment. On the phylum levels, Cyanobacteria existed in the intestinal tract of mice in CON group, while Cyanobacteria disappeared in the intestinal tract of mice in the other four groups. In addition, we also found an interesting phenomenon, that is, the relative abundance of Verrucomicrobia in the PC group increased. Studies have shown that Verrucomicrobia has a certain effect on anti-inflammation, improving glucose metabolism, and improving insulin sensitivity. The increase of Verrucomicrobia inhibits the growth of certain pathogens [54], which helps to elucidate the ameliorative effect of DMBG on T2DM. On the genus level, the relative abundance of *Allobaculum* increased significantly in the DM group and the HJB group. Previous studies have shown that *Allobaculum* abundance is positively correlated with the expression of the fat-digester enzyme ANGPTL4 (Angiogenin-like protein 4) [55]. The increase of the relative abundance of *Allobaculum* is conducive to the regulation of lipid metabolism in T2DM mice. In addition, we also found that broccoli can increase the relative abundance of *Odoribacter* and *Oscillospira*, and *Odoribacter* has anti-inflammatory effects and improves insulin resistance and glucose tolerance [56]. Wei et al. found that high-fat diet increased *Oscillospira* abundance in mice [57], and Zhu et al. also found that *Oscillospira* abundance was positively correlated with the occurrence of diabetes and inflammation [58], which was consistent with our research results. However, Verdam et al. found that the abundance of *Oscillospira* in the intestine was significantly reduced in patients with diabetes [59]. On the family level, we found that the relative abundance of Lachnospiraceae and S24-7 in the intestinal tract of T2DM mice decreased, while the relative abundance of Ruminococcaceae increased. Previous studies have also shown that Lachnospiraceae and S24-7 are related to the development of diabetes [60]. The addition of broccoli in the diet can significantly reduce the relative abundance of Erysipelotrichaceae in the intestines of T2DM mice, and increase the relative abundance of Rikenellaceae. Erysipelotrichaceae is associated with intestinal inflammatory diseases and fatty liver [61]. The metabolites of Rikenellaceae contain acetate and propionate, which can reduce the accumulation of visceral fat [62]. Broccoli will affect the abundance and diversity of intestinal flora through a series of metabolic processes, which is conducive to the intestinal flora entering the “probiotic” state, thereby alleviating T2DM.

## 5. Conclusions

This study shows that dietary intake of a certain amount of broccoli can alleviate HFD + STZ-induced diabetes in mice, and there is a certain dose correlation. The higher the dose of juiced broccoli consumed by diabetic mice, the better the improvement effect on diabetes symptoms. High dose (60 mg/(kg·BW)) of juiced broccoli can significantly reduce body weight and blood sugar in diabetic mice, increase glucose tolerance, improve insulin resistance, enhance cellular antioxidant capacity, and alleviate organ tissue damage. In addition, the role of broccoli in the treatment of diabetes also includes regulating lipid metabolism, reducing inflammation and maintaining the balance of intestinal flora. Our findings not only confirm the improving effect of broccoli on diabetes, but also provide a theoretical basis for the further application of broccoli in the prevention and treatment of diabetes and related metabolic syndrome. Exploring the specific mechanism of juiced broccoli in lowering blood sugar, regulating lipid metabolism, improving insulin resistance and alleviating inflammation is of great significance in elucidating broccoli in the prevention and treatment of T2DM. Further studies will be conducted in the future. In addition, targeted clinical trials can also provide more powerful evidence for the application of broccoli in the prevention and treatment of diabetes. Broccoli has certain preventive and palliative effects on T2DM, but its active ingredients are easy to be lost in the process of storage, transportation, cooking and digestion and absorption. These factors lead to the low bioavailability of broccoli. We will also carry out more practical research in the future to develop anti-diabetic drugs or health food.

## Data Availability

The data presented in this study are available on request from the corresponding author. The data are not publicly available due to privacy constraints.
